# Acylcarnitines profile best predicts survival in horses with atypical myopathy

**DOI:** 10.1371/journal.pone.0182761

**Published:** 2017-08-28

**Authors:** François Boemer, Johann Detilleux, Christophe Cello, Hélène Amory, Christel Marcillaud-Pitel, Eric Richard, Gaby van Galen, Gunther van Loon, Laurence Lefère, Dominique-Marie Votion

**Affiliations:** 1 Biochemical Genetics Laboratory, CHU Sart Tilman, University of Liege, Liege, Belgium; 2 Department of animal Productions: Biostatistics, Economy and animal selection, Fundamental and Applied Research for Animals & Health (FARAH), Faculty of Veterinary Medicine, University of Liege, Liege, Belgium; 3 Equine Pole, Fundamental and Applied Research for Animals & Health (FARAH), Faculty of Veterinary Medicine, University of Liege, Liege, Belgium; 4 Réseau d’EpidémioSurveillance en Pathologie Equine (RESPE), Caen, France; 5 Normandie Université, UNICAEN, Labéo Frank Duncombe, Caen, France; 6 Large Animal Internal Medicine, Gent University, Gent, Belgium; Faculty of Animal Sciences and Food Engineering, University of São Paulo, BRAZIL

## Abstract

Equine atypical myopathy (AM) is caused by hypoglycin A intoxication and is characterized by a high fatality rate. Predictive estimation of survival in AM horses is necessary to prevent unnecessary suffering of animals that are unlikely to survive and to focus supportive therapy on horses with a possible favourable prognosis of survival. We hypothesized that outcome may be predicted early in the course of disease based on the assumption that the acylcarnitine profile reflects the derangement of muscle energetics. We developed a statistical model to prognosticate the risk of death of diseased animals and found that estimation of outcome may be drawn from three acylcarnitines (C2, C10:2 and C18 -carnitines) with a high sensitivity and specificity. The calculation of the prognosis of survival makes it possible to distinguish the horses that will survive from those that will die despite severe signs of acute rhabdomyolysis in both groups.

## Introduction

Equine atypical myopathy (AM) is caused by hypoglycin A intoxication resulting from the ingestion of seeds or seedlings of certain *Acer* tree species, principally *Acer pseudoplatanus* (sycamore maple) in Europe [1–3]. Ingested hypoglycin A is metabolized into methylenecyclopropyl acetic acid-CoA (MCPA-CoA), a potent inhibitor of acyl-CoA dehydrogenases. In the mitochondria, acyl-CoA dehydrogenases catalyse the initial step in each cycle of fatty acid *β*-oxidation and their reduced activity leads to accumulation of acyl-CoAs in the mitochondria. These acyl-CoAs may then be scavenged into acylcarnitines which subsequently leave the mitochondrion and reach the peripheral circulation leading to, in AM affected horses, a characteristic blood acylcarnitine profile consistent with a multiple acyl-CoA dehydrogenase deficiency (MADD) phenotype [4]. Alternatively, MCPA may esterify with carnitine, forming MCPA-carnitine. Both hypoglycin A and MCPA-carnitine have been found in the serum of horses suffering from AM [2, 5–7].

Atypical myopathy has a high fatality rate with about three-quarter of affected horses dying with a mean survival time of less than two days [8]. Although the aetiology of AM has been uncovered, no antidote currently exists, but supportive treatment may be guided by knowledge of the underlying pathophysiological process [9, 10]. Treatment is costly, labour-and resource-intensive, and although treatment significantly increases an animal’s probability of survival, outcome is currently difficult to predict on initial assessment of a case [11]. Medical treatment is warranted in selected cases where owners are motivated, but owners may first wish to know their horses’ chances of survival and of return to normal sport capacity. From a large survey of European cases, we have learned that survival is not associated to a major risk of sequelae [8].

To determine the prognosis, veterinarians have four major steps in their clinical approach: collection of history, clinical examination, routine laboratory analyses and complementary examinations. Current knowledge concerning prediction of survival is mostly issued from a large-scale epidemiological study performed at a time when the cause was unknown; out of history, signalment, clinical variables and routine clinical pathology, few clinical signs and no biochemical parameters have been identified as predictive of survival [11]. In a more recent study focused solely on UK, a few clinical signs and high serum creatine kinase (CK) activity were associated with reduced odds of survival but only recumbency was retained in multivariate analysis [12]. Owing to recent developments, complementary examinations include the quantification of hypoglycin A [6, 13, 14] and MCPA-carnitine in blood and/ or urine samples [5]. However, only a few laboratories offer testing for the hypoglycin A toxin and its principal metabolite, MCPA-carnitine.

We then hypothesized that, although AM survivors and non survivors are clinically almost similar on presentation, outcome may be predicted early in the course of disease based on serum acylcarnitines concentrations as these metabolites indicate the severity of dysfunction of mitochondrial *β*-oxidation. The aim of this study was to set up a statistical model in order to assess the survival prognosis at early stage of the disease.

## Material and methods

### Horses and sample collection

Blood samples collected from AM cases by the principal investigator between autumn 2006 and spring 2015 were used in this study. The tentative diagnosis of AM was based on previously documented inclusion criteria for the disease [8] which was subsequently confirmed by the identification of a MADD phenotype following determination of the acylcarnitines profile.

Case histories were obtained via a standardized questionnaire available on the Internet (www.myopathie-atypique.be) and/ or via owner interviews. Data on clinical signs were obtained from medical records. The first signs noted where those considered in this study.

Through routine practice, in the aim to establish a diagnosis and to design a supportive treatment, samples were collected from the jugular vein either at pasture by the referring practitioner (n = 9) or on admission at hospital (n = 42). Total duration of the blood sampling procedure did not exceed 1 min, and non-painful sampling was confirmed by the absence of any retreat behaviour of these critically-ill animals during the procedure. Blood was centrifuged immediately; the serum was separated and stored at -80°C until analysis. It is important to underline that the samples taken the more precociously in the disease process on AM cases have been those considered in this study. Information on administered treatments and clinical outcome were extracted from clinical records and added to our database. Determination of the acylcarnitine profile and the detection of hypoglycin A were conducted with the owner's consent in order to confirm the tentative diagnosis of atypical myopathy.

All hospitalized AM horses received supportive treatment consisting mainly of intravenous fluid therapy with isotonic poly-ionic fluids and glucose solutions with concurrent insulin administration, and supplementation of vitamins, antioxidants, and/or carnitine, and oral carbohydrates.

We have asked advice from Liege University's Animal Ethics Committee who has informed us that legal restrictions do not apply because no animals were handled specifically for this experiment. The procedures performed in this study are non-experimental and routine veterinary practices with livestock species (no laboratory animals).

### Routine laboratory analyses

Serum activities of CK, creatinine, total and ionized calcium (Ca_Tot_ and Ca^2+^, respectively), electrolytes, total protein, haptoglobin, cardiac troponin I, serum amyloid A, triglycerides, glucose and lactate were determined by standard laboratory methods as well as complete blood cell count, haematocrit and arterial partial pressures in O_2_ (PaO_2_) on blood sampled on tubes with anticoagulant.

### Complementary biochemical examinations

Serum acylcarnitines concentrations were determined by tandem mass spectrometry [15]. Briefly, serum proteins were precipitated with a methanol solution containing labelled internal standards. Supernatants were evaporated under nitrogen stream and derivatized with butanolic-HCl. Butylated samples were analysed with a TQ5500 mass spectrometer (Sciex, Framingham, MA, USA). Reference ranges for acylcarnitines were derived from serum samples from 44 non-exposed healthy adult horses of various breeds and ages.

Quantification of hypoglycin A in defrosted blood sera was performed using a recently validated methodology using an aTRAQ kit for amino acid analysis of physiological fluids (Sciex, Framingham, MA, USA) [7, 16].

### Statistical analyses

Explanatory variables were grouped in 4 groups: historical data, results of physical examination, biochemical and haematological findings and, concentrations of free carnitine, acylcarnitines (short chain, medium chain and long chains) and hypoglycin A in serum. Statistical analyses were performed for each group separately.

In the first part of the analyses, chi-square and Student *t* tests were used to compare proportions and means between deceased and survivor horses, respectively. Kolmogorov-Smirnov tests were used to test the assumption that continuous explanatory variables were normally distributed. When necessary, they were transformed (Box Cox) so they follow the normal distribution.

All *P* values were corrected for multiple comparisons. However, results were only considered as indicative because Bonferroni correction tends to be too conservative.

In the second part of the analyses, PLS regression was used to handle the problem of the large number of collinear variables measured on a limited number of animals. The method also deals with missing data (EM algorithm). Five PLS components were considered and variables within these components were selected on the basis of their variable importance in the projection (VIP) and magnitude of the regression coefficients. Regression coefficients are for centred and standardized variables (proc PLS)

Variables selected to be included in the final model were those significant in the first part of the analyses (*P* ≤ 0.05), with a VIP > 1.0 and a regression coefficient greater than 0.15. We set this strict criterion because of the large number of variables initially available.

The final model is a logistic regression based on which it was possible to compute the ROC curves [17] and threshold with the best sensitivity and specificity (highest Youden index).

## Results

### Individual horses, history and sample collection

During the study period, 51 horses suspected of AM and for which blood samples were available were confirmed to be AM cases based on a MADD phenotype. From these 51 horses, 40 deceased or were euthanized (AM-D group) and 11 survived (AM-S group). Most animals were treated at the faculty of veterinary medicine of Liege or Ugent University (Belgium); seven non survivors and two survivors were treated at their home premises. Blood samples of horses treated at pasture were collected by practitioners on site.

In the AM-D group, 80% of horses were euthanized. After 48 hours, 72.5% of the AM-D group was no longer alive. The duration of disease differed significantly between both groups (2.38±0.25 days versus 12.00±1.48 days in the AM-D and AM-S group, respectively).

Demographic data are shown in Table 1. Survivors were significantly older than non survivors and frequency distribution of ages indicated that 50% of AM-S were 7 years old or older whereas 64% of AM-D were 4 years old or younger. No significant differences were found among other demographic data. Following Bonferroni correction, the mean age and duration of disease remained significantly different between both groups.

**Table 1:** Mean age and frequency distribution of demographic data of horses with atypical myopathy.

	*Non survivors* *(n = 40)*	*Survivors* *(n = 11)*
Age (years ±SEM)	4.7±0.6	8.9±1.9*
Type		
*Saddle horses*	78%	64%
*Ponies*	20%	27%
*Draught horses*	3%	9%
Sex		
*Fillies and mares*	58%	55%
*Colts and stallions*	18%	9%
*Geldings*	25%	36%
Body condition		
*Thin*	0%	10%
*Normal weight*	94%	80%
*Overweight*	6%	10%
Use for work	36%	43%

Abbreviation: SEM = standard error of the mean

* significantly different with a *P* value ≤ 0.05

### Clinical signs

Frequency distributions of clinical signs are shown in Table 2. Horses of the AM-S more frequently showed signs of muscle tremors (localized or generalized) and more often remained in standing position. Half of horses of the AM-D group were in severe pain whereas horses of the AM-S group often didn’t show signs of pain. Oesophageal obstruction and/or dysphagia were more frequently observed in the AM-D group.

**Table 2:** Frequency distribution of clinical signs in horses with atypical myopathy.

Clinical signs	*Non survivors* *(n = 40)*	*Survivors* *(n = 11)*
	Relative frequency	
Pigmenturia	94%	100%
Stiffness	76%	90%
Depressed mental status	76%	70%
Weakness	74%	90%
Want to eat	70%	71%
Trembling	69%	100%**
Recumbency	**63%**	44%
Sweating	**65%**	78%
Pain		
*Severe*	50%	17%
*Medium*	35%	33%
*Slight*	5%	0%
*No pain*	10%	50%**
Remaining standing	38%	70%*
Signs of colic	31%	40%
Oesophageal obstruction and/or dysphagia	33%	10%*
Anorexia	**10%**	11%

**Comments:** the clinical signs highlighted in bold are those that were previously found to be of prognostic value for non survival (in bolt in the non survivors column) or survival (in bolt in the column of survivors) in a large European survey [11].

* significantly different with a *P* value ≤ 0.05

** significantly different with a *P* value ≤ 0.01

Clinical examination results are reported in Tables 3 and 4. The AM-D group more frequently showed cyanotic mucous membranes whereas capillary refill time was increased in both groups. Even though the mean rectal temperature was normal in both groups, AM-S horses were more frequently hyperthermic. Respiratory difficulties (*i*.*e*., tachypnoea and expiratory dyspnoea) were equally seen in both groups. Heart murmurs were heard in both groups but arrhythmias were only detected in the AM-D group. Horses of the AM-S group had normal or diminished gut sounds whereas complete absence of gut sounds occurred in the AM-D group. None of the above variables remained significant following Bonferroni correction; therefore no variables related to clinical examination fulfilled the criteria to be included in a logistic regression to predict the prognosis. So, AM survivors and non survivors were almost clinically similar on presentation.

**Table 3:** Results of the clinical examination of horses with atypical myopathy.

Parameters Relative frequency (%)	*Non survivors* *(n = 40)*	*Survivors* *(n = 11)*
Mucous membranes		
*Normal*	14%	**22%**
*Congested*	69%	78%
*Cyanotic*	11%	0%**
*Icteric*	6%	0%
Temperature		
*Normothermia (37–38*.*5°C)*	76%	**56%**
*Hypothermia (< 37°C)*	24%	33%
*Hyperthermia (> 38*.*5°C)*	0%	11%**
Type of respiration		
*Normal rate (< 15 Breaths*.*min*^*-1*^*)*	23%	22%
*Tachypnoea (≥ 15 Breaths*.*min*^*-1*^*)*	**77%**	78%
*Dyspnoea*	**55%**	55%
Abnormal cardiac auscultation		
*Murmur*	32%	40%
*Arrhythmia*	10%	0%*
*Tachycardia (≥ 45 Beats*.*min*^*-1*^*)*	**92%**	90%
Gut sounds		
*Absent*	18%	0%**
*Diminished*	52%	50%
*Normal*	28%	50%
*Increased*	3%	0%
Miscellaneous		
*Full bladder on palpation*	58%	50%
*Abnormal rectal examination*	33%	29%
*Defaecation*	71%	**50%**

**Comments:** the clinical signs highlighted in bold are those that were previously found to be of prognostic value for non-survival (in bolt in the non survivors column) or survival (in bolt in the column of survivors) in a large European survey [11]

* significantly different with a *P* value ≤ 0.05

** significantly different with a *P* value ≤ 0.01

**Table 4:** Results of the clinical examination of horses with atypical myopathy.

Parameters (Mean ±SD)	Normal range	*Non survivors* *(n = 40)*	*Survivors* *(n = 11)*
Rectal temperature (°C)	37–38.5	37.3±0.1	37.2±0.2
Respiratory rate (Breaths.min^-1^)	<15	25.3±1.9*	24.9±2.4*
Heart rate (Beats.min^-1^)	<45	62.8±2.2*	62.8±3.8*
Capillary refill time (s)	≤2	2.8±0.1*	3.0±0.2*

Abbreviation: SD = standard error of the mean

* Value outside of reference range.

### Routine laboratory analyses

Laboratory findings are presented in Table 5. Several biochemical and haematological parameters were outside the laboratory’s reference range in both groups, but the AM-D group differed from the AM-S by higher CK activities, lower sodium concentration, lower total calcium (Ca_Tot_) and higher total white blood cell counts. One AM-D horse had a mildly increased CK (800 IU/L) only; the horse in question was sampled three hours before onset of clinical signs as it was a pasture companion of another case. For this reason, we elected to remove this horse’s CK from the dataset; this had no effect on results of statistical analyses. Following Bonferroni correction, only Ca_Tot_ remained significantly different between both groups.

**Table 5:** Biochemical and haematological findings in horses with atypical myopathy (Mean ±SD).

Parameters	Normal values	*Non survivors*	No. of dosages (max 40)	*Survivors*	No. of dosages (max 11)
***Muscle enzymes***					
CK (IU/L)	50–200	255,586±37,282*	40	126,877±41,879*^,^^a^	11
***Renal functions and ions***					
Creatinine (μmol/L)	44–140	118±10	25	94±12	8
Sodium (mmol/L)	130–145	135±1	30	141±1^b^	7
Potassium (mmol/L)	3.0–4.7	3.93±0.10	28	4.04±0.19	7
Chloride (mmol/L)	95–110	94±1*	27	97±2	7
Ca_Tot_ (mmol/L)	2.50–3.40	2.34±0.04*	19	2.72±0.06^b^	7
Ca^2+^ (mmol/L)	> 1.50	1.24±0.06*	14	1.37±0.04*	4
***Proteins ***					
Total protein (g/L)	57–69	73±2*	30	79±3*	9
Haptoglobin (mg/L)	< 500	1,702±116*	20	1,416±167*	7
Troponin I (ng/L)	10–30	4,121±984*	21	3,146±1,538*	7
Serum amyloid A (mg/L)	< 5.0	293±24*	18	303±43*	7
***Energetic metabolism***					
Triacylglycerols (mmol/L)	< 0.97	2.55±0.28*	28	1.95±0.54*	8
Glycaemia (mmol/L)	3.33–5.55	10.68±0.76*	16	9.01±0.99*	6
Lactate (mmol/L)	< 4	6.5±0.7*	23	5.1±1.3*	7
***Haematology***					
Hematocrit (%)	32–48	47±1	31	46±2	8
Total white blood cells (10^9^/L)	6–12	12.5±0.5*	23	9.3±0.6^a^	5
***Arterial blood***					
PaO_2_ (mmHg)	> 85	86±3	15	91±3	3

Abbreviations: CK, creatine kinase; Ca_Tot_ and Ca^2+^, total and ionized calcium, respectively; PaO_2_ arterial partial pressures in O_2_; SD = standard deviation

^a^ Superscripts indicate than the mean was statistically higher in non survivors *versus* survivors (*t* student or chi-square; *P* ≤ 0.05).

^b^ Superscripts indicate than the mean was statistically lower in non survivors *versus* survivors (*t* student or chi-square; *P* ≤ 0.05).

* Value outside of reference range.

### Ancillary biochemical parameters

**Fig 1:**
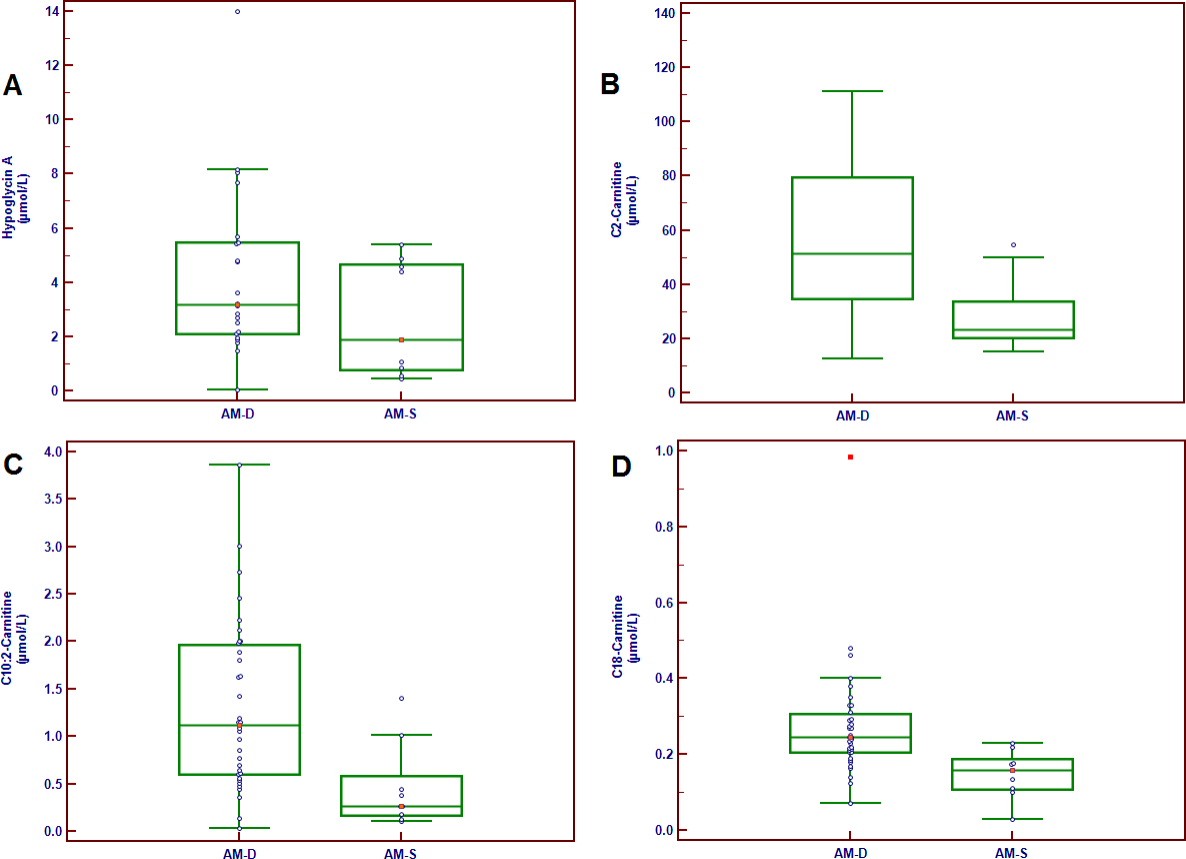
Box plot of (a) hypoglycin A, (b) C2-Carnitine; (c) C10:2-Carnitine, and (d) C18-Carnitine concentrations in sera of atypical myopathy horses among them 11 survived and 40 deceased (hypoglycin A was only measured on 9 survivors and 22 deceased horses). Comments: The central box represents the values from the lower to upper quartile (25 to 75 percentile). The middle line represents the median. A line extends from the minimum to the maximum value, excluding “outside” values (value that is smaller/higher than the lower/upper quartile ± 1.5 times the interquartile range) or “far out” values (value that is smaller/higher than the lower/upper quartile ± 3 times the interquartile range) which are displayed as separate points or red marks, respectively.

Hypoglycin A was detected in serum samples of all horses affected with AM. There was no significant difference in serum levels between survivors and non survivors (4.25±0.49 μmol/L in deceased horses versus 2.67±0.63 μmol/L in survivors; mean ±SD; Fig 1). Hypoglycin A was not found in any blood sample taken from 8 control horses (absence of chromatographic peak).

As presented in Table 6, acylcarnitine profiles were sorted into 3 groups: short, medium and long chain acylcarnitines, corresponding to the metabolic pathways in which they are involved. Short chains acylcarnitines are markers of both short chain fatty acids *β*-oxidation and some amino acids catabolism. Medium chains are markers of medium chain fatty acids *β*-oxidation while long chains are the reflection of both long chain fatty acids *β*-oxidation and long chain fatty acids transport into mitochondria (*i*.*e*. carnitine palmitoyltransferase I and II,…). Mean serum concentrations of all acylcarnitines of the AM-D and AM-S groups were outside percentile 97.5 of reference ranges obtained from healthy control horses. Based on the results, acylcarnitines were classified into 2 subcategories, namely:

- Acylcarnitines which were outside of reference ranges in both AM-S and AM-D but with no significantly different mean serum concentration between the two groups (C3, C6, C8, C10, C10:1, C12, C12:1, C14, C14:1, C16 and C16:1 -carnitine).
- Acylcarnitines which were outside of reference ranges in both AM-S and AM-D and with a mean serum concentration significantly higher in AM-D versus AM-S horses (C2, C3DC, C4, C5, C5-OH, C5DC, C8:1, C10:2, C18 and C18:1 -carnitine).

**Table 6:** Serum (mean ±SD) concentrations of free carnitine and acylcarnitines (μmol/L) in horses with atypical myopathy and control horses.

	Control horses	Horses with AM *Non survivors*	Horses with AM *Survivors*
	(n = 44)	(n = 40)	(n = 11)
**Free carnitine**			
Free carnitine	26.02±1.07	87.04±7.95*	74.41±18.07*
**Short chain acylcarnitines (C2 to C5)**			
C2-Carnitine	8.77±0.67	55.23±4.24*	36.94±9.28*^,^^a^
C3-Carnitine	0.56±0.03	2.92±0.27*	2.21±0.52*
C3DC-Carnitine	0.03±0.00	0.34±0.04*	0.11±0.02*^,^^a^
C4-Carnitine	0.36±0.02	26.62±3.67*	12.96±4.06*^,^^a^
C5-Carnitine	0.21±0.01	24.71±3.20*	9.32±2.84*^,^^a^
C5-OH-Carnitine	0.06±0.00	0.56±0.07*	0.25±0.05*^,^^a^
C5DC-Carnitine	0.18±0.01	2.20±0.28*	1.10±0.25*^,^^a^
**Medium chain acylcarnitines (C6 to C10)**			
C6-Carnitine	0.04±0.00	5.40±0.81*	2.93±1.29*
C8-Carnitine	0.02±0.00	1.45±0.18*	1.41±0.67*
C8:1-Carnitine	0.09±0.01	1.26±0.15*	0.44±0.15*^,^^a^
C10-Carnitine	0.02±0.00	0.76±0.09*	1.01±0.51*
C10:1-Carnitine	0.05±0.01	0.67±0.07*	0.44±0.07*
C10:2-Carnitine	0.03±0.00	1.31±0.14*	0.46±0.13*^,^^a^
**Long chain acylcarnitines (C12 to C18)**			
C12-Carnitine	0.04±0.01	0.28±0.03*	0.38±0.17*
C12:1-Carnitine	0.02±0.00	0.23±0.03*	0.37±0.17*
C14-Carnitine	0.03±0.00	0.21±0.02*	0.25±0.11*
C14:1-Carnitine	0.03±0.00	0.32±0.04*	0.48±0.23*
C16-Carnitine	0.14±0.04	0.68±0.07*	0.52±0.18*
C16:1-Carnitine	0.02±0.00	0.24±0.03*	0.17±0.08*
C18-Carnitine	0.07±0.01	0.28±0.02*	0.15±0.02*^a^
C18:1-Carnitine	0.04±0.01	0.57±0.06*	0.30±0.12*^,^^a^

Abbreviation: SD = standard deviation

*Mean outside percentile 97.5 of reference range obtained with control horses

^a^ Superscripts indicate than the mean was statistically higher in non survivors *versus* survivors (*t* student or chi-square; *P* ≤ 0.05).

Acylcarnitines from the first category can be used to confirm a diagnosis of AM but cannot discriminate survivors versus non survivors. The same is true for free carnitine. Acylcarnitines from the second category can also be used to confirm a diagnosis of AM but in addition they are informative regarding prognosis.

### Survival prognosis

Best prognostic factors in each of the four major steps of the clinical approach are listed in Table 7.

**Table 7:** Best prognostic predictors in the four principal components of a clinical approach.

Parameters	Probability	AUC	Sensitivity	Specificity
**In history**				
Age	if age < 5.25 years = 87.1% chance of dying	0.79	0.64	0.67
**In results of clinical examination**				
None				
**In routine laboratory analyses**				
Ca_Tot_	if < 2.69 mmol/L = 70.0% chance of dying	0.89	0.64	0.92
**In complementary biochemical examinations**				
C2	if > 17.31 μmol/L = 80.4% chance of dying	0.74	0.82	0.07
C10:2	if > 0.11 μmol/L = 80.9% chance of dying	0.84	0.73	0.15
C18	if > 0.21 μmol/L = 92.6% chance of dying	0.86	0.18	0.37

Abbreviation: AUC = area under the curve

Horses younger than 5.25 years have 87.1% chance of dying. This threshold of 5.25 years yields the highest sensitivity (SE) of 64% and specificity (SPE) of 67%. Horses that were still alive at day 3 from onset of clinical sings have 50% chance of surviving.

The Ca_Tot_ is the most relevant routine laboratory marker to predict prognosis. Affected animals with Ca_Tot_ lower than 2.69 mmol/L have a 70% chance of dying. This threshold yields the best SPE (92%) and SE (64%).

According to partial least square (PLS) and logistic regressions, C2, C10:2 and C18–carnitine appeared to be the best parameters to predict survival among the different markers selected (Table 7 and Fig 1). Neither Ca_Tot_, nor age, nor hypoglycin A were retained as significant markers by the algorithm.

The model that best predicted probability of AM horses survival is:

$$Logit(p)=b_{0}+b_{1}\;C2-Carnitine+b_{2}\;C10:2-Carnitine+b_{3}\;C18-Carnitine$$

where

b0 = 5.38 (standard error: 2.31)
b1 = -0.06 (standard error: 0.03)
b2 = -0.55 (standard error: 1.01)
b3 = -18.94 (standard error: 9.61)
C2, C10:2 and C18 -Carnitine units: μmol/L

**Fig 2:**
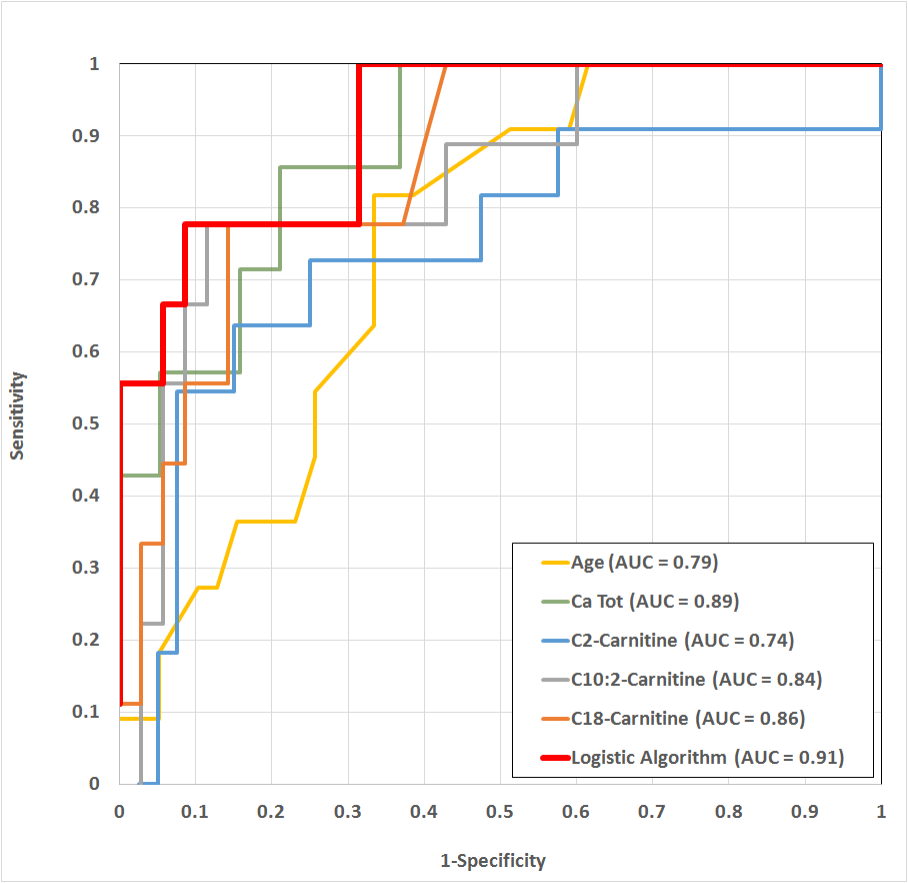
ROC curves comparison (with their corresponding AUC) for age, Ca_Tot_, C2-carnitine, C10:2-Carnitine, C18-Carnitine and the logistic algorithm in the estimation of survival prognosis. Abbreviation: AUC = area under the curve.

Compared to each individual marker, the corresponding ROC curves demonstrate that the logistic algorithm provides the best sensitivity and specificity, with an area under the curve (AUC) rising to 0.91 (Fig 2).

This multivariate approach has subsequently been validated against independent AM animal’s cohorts. Acylcarnitines have been quantified on eight AM horses from Denmark (4 survivors and 4 deceased) [18] and four animals from New-Zealand (2 survivors and 2 deceased) [19]. Except for the case with mildly elevated CK that was collected before symptoms development, all samples were collected the more precociously after symptoms apparition to analyze acylcarnitines profile. Concentrations of C2, C10:2 and C18 -Carnitine have been then introduced in the logistic equation to predict their outcome. Estimation of survival probability observed for these animals was in agreement with our expectations: survivors and deceased equines were respectively classified with high and low probability of survival (Table 8).

**Table 8:** Survival prognosis estimated on independent cohort of horses with atypical myopathy [17, 18]. Prognosis varies from 0 to 1; the closer the value is to zero, the higher the risk of mortality. Conversely, the closer the value approaches 1, the greater the chance of survival. Low mortality risk is marked in bold.

Animal with atypical myopathy	Survival probability
Denmark—AM-S—1	**0.512**
Denmark—AM-S—2	**0.985**
Denmark—AM-S—3	**0.836**
Denmark—AM-S—4	**0.250**
Denmark—AM-D—1	0.000
Denmark—AM-D—2	0.013
Denmark—AM-D—3	0.024
Denmark—AM-D—4	0.026
New-Zealand—AM-S—1	**0.847**
New-Zealand—AM-S—2	**0.975**
New-Zealand—AM-D—1	0.001
New-Zealand—AM-D– 2	0.011

Abbreviations: AM-S: horse suffering from atypical myopathy who survived; AM-D: horse suffering from atypical myopathy who deceased or was euthanized

## Discussion

In this study, factors that enable differentiation between AM cases that are unlikely and those that are likely to survive were identified. Prognostic factors were sought among the four pivotal steps of most clinical approaches, *i*.*e*. historical data, clinical examination, routine clinical pathology and ancillary testing.

The only historical risk factor associated with a fatal outcome in our study was a young age, which has previously been found to be associated with an increased risk of being affected by AM. [20] This finding suggests that younger individuals have a higher sensitivity to the toxin. As atypical myopathy results from a muscle energetics imbalance, the higher susceptibility of youngsters might be a reflection of higher energetic needs in growing horses.

In a large exploratory data analysis of European cases, negative prognostic factors were recumbency, sweating, anorexia, tachycardia, tachypnoea and dyspnoea [11]. Positive prognostic factors were: remaining standing most of the time, normothermia, normal mucous membranes, presence of defaecation, and vitamin / antioxidant therapy [11]. In the current study, all horses received similar supportive treatment, therefore the effect of treatment could not be assessed but remaining standing up, normal rectal temperature, normal mucous membrane colour and presence of digestive sounds were also found to be associated with a good prognosis. In addition, other clinical parameters were identified as positive prognosis factors such as the absence of signs of pain, a lower frequency of oesophageal obstruction and/or dysphagia and absence of cardiac arrhythmias. As none of the clinical parameters, in the present study, remained significantly different following Bonferroni correction, they cannot be used to predict prognosis with any certainty and should be used with caution in clinical decision-making.

However, it can be stated that the longer the horse survives, the more the outcome becomes positive. Therefore, it may be warranted in future cases attempt to treatment, for at least two days from onset of clinical signs if there is no considerable pain or cardiovascular compromise (such as indicated by tachycardia, cardiac arrhythmias and/ or cyanotic mucous membranes) or when they can be reverted by the initial supportive therapy, and if the horse can be fed with a diet rich in carbohydrates in the absence of oesophageal obstruction and/or dysphagia. Recommended supportive therapy for AM is extensively described elsewhere [9, 10]. However, if respiratory difficulties arise or progressively worsen euthanasia may be indicated [21, 22]. In our AM-D group, horses were euthanized because of severe expiratory dyspnoea and/ or compromised gas exchanges as indicated, when measured, by low arterial partial pressures in O_2_ and increased arterial partial pressures in CO_2_.

In this study, severe biochemical disorders were found. In particular, massive muscle cell destruction was associated with electrolyte abnormalities and high CK activities in serum. The determination of Ca_Tot_ has predictive value for survival. The hypocalcaemia frequently found in AM-affected hoses might contribute to dysrhythmias observed in some horses [23]. Intravenous calcium supplementation may be warranted to maintain or restore serum ionized calcium concentration in AM-affected horses [9].

In rat, hypoglycin A is rapidly metabolized *in vivo* [24] and may be undetectable in biological samples. The same is true for human patients suffering from Jamaican vomiting sickness [25], a disease resulting from the ingestion of unripe ackee fruit which contains hypoglycin A [26]. The kinetics of hypoglycin A in horses are currently unknown but high levels have been detected in apparently healthy horses [1, 5]. Also, a wide range of hypoglycin A concentrations was found both in the group of AM-D and AM-S horses whereby mean serum concentration was not significantly different between the two groups. Therefore, detection of hypoglycin A in serum can be considered a biomarker of exposure but has no prognostic value.

Metabolism of hypoglycin A produces the toxic metabolite MCPA which may form ester with carnitine but also with glycine, two important detoxification forms which are excreted in urine [27]. Recently, a method allowing the determination of acylcarnitines and MCPA conjugates in small samples of serum and urine has been validated [6]. The level of MCPA-conjugates might contribute to both diagnosis and prognostication of AM and our predictive model might be further improved by including MCPA-carnitine concentration. MCPA-glycine which is more readily excreted in the urine is less likely to be a valuable serum parameter for estimation of survival. It is unknown which serum concentrations of MCPA need to be reached to induce signs of AM. Since subclinical cases occur [5, 21, 28], we hypothesise that there is a dose-dependent action similar to other species [29] and that the toxic threshold may vary depending on the current energy balance of the horse.

MCPA-CoA inhibits short and medium chain acyl-CoA dehydrogenases [30–34] and reduces several cofactors, such as coenzyme A and carnitine, which are essential to the *β*-oxidation of long-chain fatty acids thus inhibiting their transport into the mitochondria [35]. Consequently, fatty acids conjugated with carnitines accumulate in the serum. In horses, as obtaining a blood sample is easier than urine collection and because long chain acylcarnitines are not excreted in urine, it was therefore decided to search for the best prognostic biomarkers in blood among short, medium and long chain acylcarnitines thus covering the whole process associated with alteration of the *β*-oxidation of fatty acids. Also, acylcarnitines profiling is feasible in a large numbers of laboratory facilities, while MCPA-conjugates may only be analysed in a few laboratories that have developed specific methods for diagnosis of hypoglycin A intoxication.

The striking finding of this study is the ability to specify a prognosis based on early blood testing, at the start of the clinical examination by the practitioner or even at a sub-clinical state as shown by one horse that was sampled 3 hours before clinical signs became apparent and which died as would have been predicted based on its serum levels of C2, C10:2 and, C18 -Carnitine (with a prognosis for survival estimated at 0.3%). Considered individually, C2-Carnitine has good sensitivity for determination of both diagnosis and prognosis, but it is unfortunately a very unspecific marker of ketosis. C10:2-Carnitine is increased in 2,4-dienoyl-CoA reductase deficiency while C18-Carnitine reflects either translocase or carnitine palmitoyltransferase II. Considered all together, these three parameters are unspecific markers of MADD but revealed the highest sensitivity in mortality prediction.

To date, no therapy is effective to cure AM and care is essentially supportive. Many animals die during the course of intoxication and euthanasia often appears as the ultimate veterinary’s expedient. However, some intoxicated horses survive despite a dramatic initial clinical picture. In this context, survival prognosis could be of critical interest to prevent euthanasia. Indeed, intensive care may be focused on animals with good survival expectancy while individuals with high death probability could be sacrificed more “pragmatically” when there are signs of suffering that cannot be suppressed. Prognosis of survival may additionally be useful when testing new therapies, in order to evaluate their efficacy. Effect of any new treatment should be relativized if the new drug appears only effective in animals with an initial high predictive value of survival. Contrarily, the efficacy of any new protocol could be highly consolidated if mortality prognosis is initially elevated.

Based on the results of this study, we recommend practitioners to pose an initial tentative diagnosis of AM based on history (*i*.*e*. acute signs of rhabdomyolysis in horses at pasture with possible hypoglycin A ingestion), clinical examination (*i*.*e*. pigmenturia being the most specific but not pathognomonic signs) and routine laboratory tests (*i*.*e*. CK activities but also Ca_Tot_ and other biochemical parameters which are also useful to correct electrolyte imbalances). Confirmation of the diagnosis is best performed by determining acylcarnitines profile and hypoglycin A or MCPA-carnitine concentration in order to confirm exposure to the etiological agent. And finally, the best evaluation of survival prognosis is achieved by integrating the levels of C2, C10:2 and C18:1 -Carnitine into the proposed algorithm. To date, acylcarnitines profile analysis is generally performed in a research context in only few specific laboratories, without any “emergency” consideration. Our results provide however a major incentive for such laboratories to implement, in their own organization, the analysis of corresponding samples on a daily basis to provide prompt and comprehensive diagnostics.

Regardless of eventual outcome of AM, its clinical manifestations are severe signs of acute rhabdomyolysis of mostly postural and respiratory muscles and the myocardium, which all utilise fatty acids as a primary energy source. We developed a scoring system to predict survival in AM affected cases based on early blood sampling. The scoring system proposed in this study may help clinicians with management decisions including euthanasia. In further cases, we sought to apply this procedure and correlate results of this equation with outcomes.

## Supporting information

S1 Table Original dataset is available as supporting file of the manuscript. (XLSX)
